# Metabolic Rewiring in the Face of Genomic Assault: Integrating DNA Damage Response and Cellular Metabolism

**DOI:** 10.3390/biom15020168

**Published:** 2025-01-23

**Authors:** Wenjian Ma, Sa Zhou

**Affiliations:** 1 College of Biological and Chemical Engineering, Qilu Institute of Technology, Jinan 250200, China; 2 College of Biotechnology, Tianjin University of Science and Technology, Tianjin 300457, China; zhousa@tust.edu.cn

**Keywords:** DNA damage response (DDR), DNA repair, ROS, oxidative stress, cellular metabolism, cancer therapy

## Abstract

The DNA damage response (DDR) and cellular metabolism exhibit a complex, bidirectional relationship crucial for maintaining genomic integrity. Studies across multiple organisms, from yeast to humans, have revealed how cells rewire their metabolism in response to DNA damage, supporting repair processes and cellular homeostasis. We discuss immediate metabolic shifts upon damage detection and long-term reprogramming for sustained genomic stability, highlighting key signaling pathways and participating molecules. Importantly, we examine how DNA repair processes can conversely induce metabolic changes and oxidative stress through specific mechanisms, including the histone H2A variant X (H2AX)/ataxia telangiectasia mutated (ATM)/NADPH oxidase 1 (Nox1) pathway and repair-specific ROS signatures. The review covers organelle-specific responses and metabolic adaptations associated with different DNA repair mechanisms, with a primary focus on human cells. We explore the implications of this DDR–metabolism crosstalk in cancer, aging, and neurodegenerative diseases, and discuss emerging therapeutic opportunities. By integrating recent findings, this review provides a comprehensive overview of the intricate interplay between DDR and cellular metabolism, offering new perspectives on cellular resilience and potential avenues for therapeutic intervention.

## 1. Introduction

Maintaining cellular homeostasis is crucial for the proper functioning and survival of organisms. Central to this homeostasis is the preservation of genomic integrity, which is constantly challenged by various endogenous and exogenous stresses [1]. These insults can lead to DNA damage, threatening genomic stability and triggering a cascade of cellular responses known as the DNA damage response (DDR). The DDR coordinates the detection, signaling, and repair of DNA damage and is essential for preventing mutations that could lead to diseases such as cancer and neurodegenerative disorders [2].

Over the past decade, it has become increasingly clear that DDR is tightly intertwined with cellular metabolism, which provides the necessary energy and nucleotide precursors for DNA repair [3,4]. Emerging evidence suggests a complex bidirectional relationship between DDR and cellular metabolism. While it has long been recognized that metabolic processes can influence DNA integrity through the generation of reactive oxygen species (ROS) and other byproducts [5,6], recent studies have unveiled that DNA damage and its repair processes can conversely induce significant metabolic rewiring [7,8,9,10]. This crosstalk between DDR and metabolism is now recognized as a critical factor in cellular resilience and adaptation to stress [8,11]. Therefore, understanding the intricate interplay between DDR and cellular metabolism holds significant implications for human health and disease.

By integrating recent findings from molecular biology, biochemistry, and clinical research, this review seeks to provide a holistic understanding of how cells coordinate their genome maintenance and metabolic activities to ensure survival and functionality in the face of genomic stress. This knowledge is crucial for advancing our understanding of cellular physiology and for developing novel therapeutic approaches to combat diseases associated with genomic instability and metabolic dysregulation.

## 2. The DNA Damage Response: Key Pathways and Processes

The DDR encompasses a complex array of mechanisms that sense DNA damage, activate signaling cascades, and effectuate repair processes. The DDR system not only guards against mutations but also determines cellular outcomes, such as cell cycle arrest, senescence, or apoptosis, depending on the extent of the damage [12,13].

The detection of DNA damage is the crucial first step in initiating the DNA damage response (DDR). Key sensors are highly conserved across eukaryotes, from yeast to humans. In mammals, the primary sensors include ATM (ataxia telangiectasia mutated), ATR (ATM and Rad3-related), and DNA-PK (DNA-dependent protein kinase) [14]. Their yeast homologs, Tel1 and Mec1, perform similar functions [15]. These kinases coordinate with chromatin-based sensors, particularly the histone variant H2AX, which serves as a molecular scaffold for repair factor recruitment and signal amplification [16]. ATM/Tel1 primarily responds to double-strand breaks (DSBs), while ATR/Mec1 is activated by single-stranded DNA, often exposed at stalled replication forks [17]. Upon activation, both kinases initiate a signaling cascade that activates checkpoint kinases, notably CHK1 and CHK2, which halt the cell cycle to allow time for repair [14]. Recent studies have revealed that these sensors are not just responders to DNA damage but also play roles in metabolic regulation. For instance, ATM has been shown to regulate mitochondrial function and cellular ROS levels, linking DNA damage sensing directly to metabolic processes [18].

DNA repair is a tailored response that is determined by the specific type of DNA damage. Base excision repair (BER) corrects small lesions, such as those caused by oxidation or alkylation, by removing damaged bases and filling in the gap with the correct nucleotide [19]. Nucleotide excision repair (NER), on the other hand, is responsible for repairing bulky DNA adducts, such as those induced by ultraviolet (UV) light, by excising a short single-stranded DNA segment around the lesion [20]. Mismatch repair (MMR) specifically recognizes and repairs base–base mismatches and insertion/deletion loops that arise during DNA replication or recombination, thereby maintaining genome stability and preventing mutations [21]. Double-strand breaks (DSBs), one of the most dangerous forms of damage, are primarily repaired by two mechanisms: homologous recombination (HR) and non-homologous end joining (NHEJ). HR is a high-fidelity repair process that uses a sister chromatid as a template for repair, making it active predominantly during the S and G2 phases of the cell cycle [22]. In contrast, NHEJ is a quicker but more error-prone process that directly ligates the broken DNA ends without the need for a template, and it can occur throughout the cell cycle [23]. When DNA damage blocks replication fork progression and cannot be immediately repaired, cells can employ translesion DNA synthesis (TLS), a damage tolerance mechanism that allows replication to continue past DNA lesions through the use of specialized DNA polymerases. While TLS is often error-prone, it prevents prolonged replication fork stalling that could lead to more severe genomic instability [24,25]. Each of these pathways is energy-intensive and requires precise coordination between DDR signaling and metabolic processes.

The cellular response to DNA damage involves a sophisticated decision-making process that determines cell fate. When DNA damage is detected, cells initially arrest their cell cycle through the activation of checkpoints, providing time for repair [8]. If the damage is successfully repaired, cells can resume proliferation. However, when DNA damage is extensive or persistent, cells may undergo senescence, a state of permanent cell cycle arrest that prevents the propagation of potentially dangerous mutations [26]. In cases of severe DNA damage that threatens genomic integrity, cells may activate programmed cell death pathways to eliminate themselves from the population [27]. This decision-making process is tightly regulated and involves complex signaling networks that integrate information about the type and extent of DNA damage, the cell’s metabolic state, and the cellular context [28]. Recent studies have revealed that these cellular fate decisions are intimately linked with metabolic adaptations, as different outcomes (repair, senescence, or apoptosis) require distinct metabolic resources and energy investments [29,30].

## 3. Metabolic Rewiring in Response to DNA Damage

DNA repair is an energy-intensive process that requires both specific metabolites and significant ATP consumption. Cells must therefore coordinate various metabolic pathways to meet these demands while maintaining essential cellular functions. Studies using direct ROS measurements have revealed sophisticated cellular responses to DNA damage. These include mechanisms and adaptations that occur in distinct temporal phases by which cells rewire their metabolism to support DNA repair processes [31,32].

### 3.1. Nucleotide Metabolism Rewiring

The immediate requirement for DNA repair is an adequate supply of nucleotides. Cells respond to DNA damage by rapidly upregulating nucleotide synthesis and modulating nucleotide pools, which refers to the collective concentration of the four deoxyribonucleoside triphosphates (dNTPs) within a cell [33]. The regulation of dNTP pools is particularly crucial, as unbalanced dNTP levels can lead to increased mutation rates and genome instability [34]. In response to DNA damage, cells activate ribonucleotide reductase (RNR), the rate-limiting enzyme in dNTP synthesis, through multiple mechanisms including transcriptional upregulation and allosteric regulation [35]. One-carbon metabolism plays a vital role in nucleotide synthesis by providing essential carbon units through the folate and methionine cycles [36]. DNA damage triggers increased flux through these pathways to support enhanced nucleotide synthesis. This involves the upregulation of key enzymes such as serine hydroxymethyltransferase (SHMT) and methylenetetrahydrofolate dehydrogenase (MTHFD) [37]. This metabolic reprogramming is coordinated with cell cycle responses, as shown by studies revealing that ATM activation following DNA damage directly influences nucleotide synthesis pathways [38].

The nucleotide salvage pathways also become activated during DNA repair, allowing cells to recycle existing nucleotides and their precursors more efficiently. This is particularly important in non-proliferating cells, where de novo nucleotide synthesis is typically low [39]. Recent evidence suggests that the choice between de novo synthesis and salvage pathways is influenced by the type of DNA damage and the specific repair pathway engaged [40]. For example, studies in H3K27M-mutant diffuse midline glioma have shown that these tumors can utilize the purine salvage pathway to resist treatment, demonstrating that cells can adapt different nucleotide synthesis strategies depending on the context of DNA damage [41]. Furthermore, mechanistic studies have revealed that GTP metabolism plays a crucial role in coordinating DNA repair responses to genotoxic stress, highlighting the complex interplay between nucleotide synthesis pathways and DNA repair [42]. SAMHD1, a dNTP hydrolase, is recruited to DNA damage sites where it has non-catalytic roles in DNA break repair and replication fork restart through interactions with repair nucleases [43,44]. SAMHD1 deficiency can indirectly influence ribonucleotide reductase (RNR) activity through changes in dNTP levels, which allosterically regulate RNR and impact the specificity of dNTP synthesis [45]. Similarly, MTHFD2, an enzyme involved in mitochondrial folate one-carbon metabolism and nucleotide biosynthesis, has a non-catalytic role in promoting DNA break repair through direct interactions with DNA repair enzymes [46,47]. Collectively, these findings reveal that nucleotide metabolism enzymes serve dual roles in both metabolic regulation and direct DNA repair processes, establishing a sophisticated regulatory network that coordinates nucleotide availability with DNA repair needs.

### 3.2. Energy Metabolism Adaptations to Support Repair

DNA repair processes require substantial energy input, beginning with the activation of the DNA damage response itself. ATP is required for the activation of DDR kinases like ATM and ATR, as well as for the phosphorylation cascades they initiate [14]. These energy requirements continue throughout the repair process, though the ATP demands vary significantly among different repair pathways. Upon DNA damage, cells enhance glucose uptake and utilization to meet these increased ATP requirements. This involves both increased glucose transporter expression and enhanced glycolytic flux [48].

Different DNA repair pathways exhibit distinct energy requirements. Base excision repair (BER) is relatively energy-efficient, requiring ATP primarily for its final ligation step [49]. In contrast, nucleotide excision repair (NER) is highly energy-intensive, consuming ATP for multiple steps including DNA unwinding, damage verification, and excision [20]. Mismatch repair (MMR) requires ATP for several steps including mismatch recognition and strand excision [50]. Among all repair pathways, homologous recombination (HR) shows the highest energy demand, requiring ATP for extensive DNA processing, strand invasion, and DNA synthesis [22]. Non-homologous end joining (NHEJ), while still ATP-dependent, generally requires less energy as it involves direct ligation of broken DNA ends [23].

Mitochondrial function is modulated to support these varying energy demands. Studies have shown that DNA damage triggers changes in mitochondrial dynamics and activity to optimize ATP production [48]. This includes adjustments in electron transport chain activity and oxidative phosphorylation efficiency. Furthermore, cells may shift their substrate utilization patterns, often increasing glutamine consumption to support both energy production and nucleotide synthesis [51].

Beyond these direct energy-producing pathways, cells also coordinate multiple metabolic circuits to support DNA repair. The pentose phosphate pathway becomes activated to provide both ribose-5-phosphate for nucleotide synthesis and NADPH for redox balance. These various metabolic adaptations—including glycolysis, oxidative phosphorylation, glutamine metabolism, and the pentose phosphate pathway—are carefully coordinated to meet the specific demands of different repair processes. The detailed mechanisms of how these pathways are regulated, particularly their roles in maintaining redox balance and nucleotide pools, will be discussed in subsequent sections.

### 3.3. Metabolic Signaling and Regulation

The metabolic adaptations supporting DNA repair are coordinated by several key signaling pathways. Recent studies have revealed that histone H2AX, beyond its role in DNA damage sensing, plays a crucial role in metabolic regulation. Upon DNA damage, H2AX activates Nox1 through Rac1 GTPase, leading to controlled ROS production that serves as a key metabolic signal [31]. This H2AX-Nox1/Rac1 pathway represents a direct mechanistic link between DNA damage detection and metabolic adaptation. The pathway involves release of the Nox1 activator NOXA1 from its inhibitory interaction with 14-3-3zeta, allowing for precise control of ROS-mediated metabolic signaling.

AMPK, a central energy sensor, becomes activated in response to DNA damages both directly and through ROS-mediated pathways [52]. Studies have shown that AMPK can be activated by various DNA-damaging agents and subsequently phosphorylate several DDR proteins, including p53 at Ser15 [53]. When activated, AMPK helps coordinate cellular response to DNA damage by promoting cell survival pathways and metabolic adaptation, including increased glucose uptake and suppression of energy-consuming processes not essential for repair [54,55].

The activation of AMPK typically leads to the inhibition of mTOR signaling, creating a metabolic checkpoint that helps cells balance their energy resources. However, the regulation of mTOR during DNA repair is complex and context-dependent [56]. While acute DNA damage often leads to mTOR suppression to conserve energy, sustained repair processes may require specific mTOR-dependent activities to support the synthesis of repair proteins and metabolic enzymes [57].

NAD+-dependent pathways are particularly critical in coordinating metabolism with DNA repair. NAD+ serves both as a cofactor for repair enzymes and as a substrate for signaling molecules like PARP and sirtuins [58]. Recent studies in yeast have demonstrated that NER efficiency is directly linked to NAD+ availability, highlighting the integral connection between metabolic state and repair capacity [59]. The consumption of NAD+ by repair processes, especially through PARP activation during severe DNA damage, can significantly impact cellular metabolism, necessitating adaptations in NAD+ synthesis and salvage pathways. Sirtuins, particularly SIRT1, play crucial roles in linking metabolism to the DDR through their NAD+-dependent deacetylase activity. SIRT1 has been shown to deacetylate several DNA repair proteins, including XPC in nucleotide excision repair and WRN helicase, and collaborates with ATM to maintain genomic stability [60,61,62]. All these activities are dependent on NAD+ availability.

Metabolic adaptation is also coupled with the regulation of multiple transcription factors that coordinate repair and metabolic processes by altering the expression of metabolic enzymes and transporters. For example, p53 activation following DNA damage leads to increased expression of genes involved in glucose metabolism and mitochondrial function, helping establish a metabolic environment that supports DNA repair [63,64]. These transcriptional responses help establish a metabolic environment conducive to efficient DNA repair while maintaining essential cellular functions.

## 4. The Dark Side of Metabolic Adaptation: DDR-Induced Oxidative Stress

Metabolic adaptations during DNA repair, while necessary, can have detrimental effects on cellular homeostasis. Studies have shown that ROS levels are significantly increased in cells and tissues during DNA damage repair [9,10,65,66]. This is not just a side effect of repair, but represents an active signaling mechanism. DNA damage triggers ROS production through multiple pathways, with one key mechanism involving the histone variant H2AX and the NADPH oxidase Nox1 [31]. In cancer treatment, cisplatin has been shown to induce oxidative stress through multiple mechanisms including mitochondrial dysfunction and NADPH oxidase stimulation [9]. The relationship between DNA repair and ROS generation is particularly evident in DNA repair-deficient cells. For example, cells lacking XPA exhibit chronic oxidative stress due to PARP hyperactivation and NAD+/SIRT1 reduction [58]. Recent studies using a site-specific DNA double-strand break system in *Saccharomyces cerevisiae* have provided direct evidence that the DNA repair process itself triggers ROS production [65]. This was demonstrated by the finding that deletion of genes involved in homologous recombination resection stimulates ROS production, while blocking non-homologous end joining suppresses ROS levels.

The temporal dynamics of repair-induced ROS production follow a distinct pattern, with different repair pathways leading to characteristic ROS signatures [32]. For instance, single-strand break repair typically generates a rapid but transient increase in ROS, while double-strand break repair leads to a more sustained ROS elevation [9]. These different patterns may serve as specific signaling mechanisms to coordinate cellular responses to different types of DNA damage. Although the generation of ROS is a secondary event, they may function as important signaling molecules. For example, they can reversibly oxidize specific cysteine residues in signaling proteins, leading to conformational changes that alter protein function [67]. H_2_O_2_ produced during repair can oxidize and activate ATM independently of DNA damage, creating a feedback loop that amplifies the DNA damage response [38]. Beyond their role in feedback and signal amplification, repair-induced ROS can influence cell fate decisions by modulating repair pathway choice and activating stress response pathways [68]. Additionally, ROS production during repair can stimulate immune responses and influence the tumor microenvironment, which is particularly relevant in the context of cancer therapy [69].

The mechanisms underlying DDR-induced oxidative stress involve multiple cellular processes, with mitochondria playing a central role. ATM kinase, a key DNA damage sensor, has been shown to regulate both nuclear DNA damage responses and mitochondrial homeostasis through a ROS-dependent feedback loop [38]. DNA damage can trigger mitochondrial stress responses, leading to changes in electron transport chain activity and increased ROS production [70]. Furthermore, ATM deficiency results in elevated mitochondrial ROS levels and disrupted mitochondrial function [38,71].

Beyond mitochondrial sources, DDR-induced oxidative stress also stems from NADPH-dependent processes. Studies have revealed that DNA damage repair can lead to activation of NADPH oxidases (NOX), with Nox1 emerging as a crucial player. Nox1 is activated through a H2AX-dependent pathway in humans that involves the release of the Nox1 activator NOXA1 from its inhibitory interaction with 14-3-3zeta [31]. In yeast, NOX (Yno1) shows enhanced activity following DNA damage [65]. Moreover, an extensive DNA damage response can lead to hyperactivation of PARP, resulting in NAD+ depletion. This affects not only cellular redox balance but also mitochondrial function, as NAD+ is crucial for electron transport chain activity [58,72].

The consequences of repair-induced oxidative stress can create a detrimental feedback loop. While cells attempt to counteract oxidative stress through antioxidant responses, excessive ROS production can overwhelm these defenses. Studies in yeast have shown that loss of antioxidant genes like *SOD1* can lead to synthetic lethality upon DNA damage, which highlights the critical interplay between DNA repair and redox homeostasis [65,73,74]. The oxidative stress generated during repair can itself cause additional DNA damage, potentially creating a vicious cycle that amplifies genomic instability [32].

The cellular response to repair-induced oxidative stress involves sophisticated regulatory mechanisms. For instance, the Yap1 transcription factor, a key regulator of oxidative stress responses in yeast, shows distinct patterns of nuclear localization following DNA damage [32]. This nuclear accumulation occurs with different kinetics compared to direct oxidative stress, suggesting that DNA damage-induced ROS production serves specific signaling functions. Similar regulatory mechanisms have been identified in mammalian cells, where DNA damage-induced ROS can activate specific transcriptional programs through redox-sensitive transcription factors [31]. Understanding the complexity of repair-induced oxidative stress and signaling is particularly important in the context of cancer therapy, which will be discussed below.

## 5. Key Pathways and Molecules in DDR–Metabolism Crosstalk

**Figure 1:**
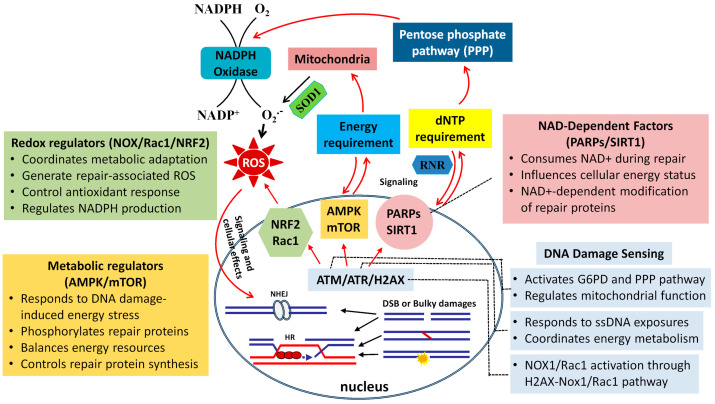
A diagram illustrating the complex interplay between DNA damage response and cellular metabolism. DNA damage in the nucleus triggers a coordinated response involving both nuclear and cytoplasmic factors to maintain genomic stability through metabolic adaptation. Following bulky DNA damage (crosslinks, DSBs, or breaks with complex ends), DNA damage sensors (ATM, ATR, etc.) initiate signaling cascades that regulate various metabolic processes. These include AMPK, which responds to energy stress and regulates repair, and mTOR, which balances resource allocation. These regulators interact with effector proteins such as PARP1 and SIRT1 (which compete for NAD+ and influence repair efficiency) and NRF2 (which coordinates antioxidant responses). Metabolic support enzymes, including G6PD and RNR, are activated to provide essential metabolites for repair, which stimulates ROS generation, potentially through activation of the pentose phosphate pathway (PPP) or mitochondrial oxidative phosphorylation. Information boxes detail the specific functions of each component. This network highlights the bidirectional nature of DDR–metabolism crosstalk.

The coordination between DNA damage response and cellular metabolism is highly sophisticated. It requires complex molecular mechanisms to sense damage, relay signals, and execute appropriate metabolic changes. Several key pathways and molecules serve as critical nodes in this complex regulatory network (Figure 1).

### 5.1. DNA Damage Sensors as Metabolic Regulators

ATM, primarily known for its role in DNA damage sensing, has emerged as a crucial regulator of cellular metabolism. Beyond its canonical role in the DNA damage response, ATM directly coordinates the cellular redox state through multiple mechanisms. Studies have shown that ATM can be activated by oxidative stress independently of DNA damage, establishing it as a redox sensor [38]. This activation occurs through formation of disulfide-crosslinked ATM dimers that are dependent on a critical cysteine residue, a mechanism distinct from its activation by DNA breaks, where ATM dimers dissociate into active monomers [38]. Active ATM stimulates glucose-6-phosphate dehydrogenase activity through direct protein–protein interaction, promoting NADPH production and enhancing cellular antioxidant capacity [66].

Similarly, ATR’s functions extend beyond checkpoint regulation into metabolic control. ATR has been found to localize to the nuclear envelope and mitochondria, where it helps maintain mitochondrial function and regulate cellular energy metabolism [75]. This spatial regulation allows ATR to coordinate cellular responses to both DNA damage and metabolic stress.

Recent studies show that ATM and ATR also function in managing DNA–RNA hybrid structures called R-loops, which represent an important intersection between transcription, DNA damage, and oxidative stress. These three-stranded nucleic acid structures, consisting of an RNA–DNA hybrid and displaced single-stranded DNA, form naturally during transcription but can become pathological when not properly resolved [76]. Recent studies have shown that oxidative stress promotes R-loop formation [77], while persistent R-loops can trigger ROS production through effects on transcription and replication stress [78]. ATM and ATR play crucial roles in this process; ATM is activated by R-loop-induced DNA damage [79], while ATR responds to associated replication stress [80]. This creates a regulatory circuit where ATM/ATR activation can both respond to and influence R-loop dynamics, connecting these structures to broader metabolic regulation through effects on transcription and oxidative stress responses.

### 5.2. Metabolic Sensors in DDR Regulation

AMPK, the primary cellular energy sensor, plays a pivotal role in coordinating metabolism with DNA repair. Upon activation by energy stress or DNA damage, AMPK phosphorylates multiple targets to enhance catabolic processes while suppressing anabolic pathways [81]. Recent studies have revealed that AMPK directly phosphorylates several DNA repair proteins, including EXO1 and H2AX, linking energy status to repair pathway choice [82].

The NAD+-dependent deacetylases, particularly SIRT1, serve as critical links between cellular metabolism and genome maintenance. SIRT1 deacetylates several key DDR proteins, including NBS1 and Ku70, in an NAD+-dependent manner [83,84]. This dependency on NAD+ allows cells to modulate DNA repair efficiency based on their metabolic state. During DNA repair, PARP activation can deplete cellular NAD+ pools, thereby affecting SIRT1 activity and creating a regulatory feedback loop [58]. The role of NAD+ metabolism in coordinating DNA repair has been shown to be particularly important in determining repair pathway choice and efficiency [31].

### 5.3. Transcriptional Integration of DDR and Metabolism

p53, beyond its role as the “guardian of the genome”, functions as a master regulator of cellular metabolism. In response to DNA damage, p53 not only activates cell cycle checkpoints but also orchestrates metabolic adaptations through regulation of genes involved in glycolysis, oxidative phosphorylation, and glutamine metabolism [64]. This coordination ensures appropriate metabolic support for DNA repair processes. Specifically, p53 activation can enhance mitochondrial respiration while simultaneously limiting glycolysis, helping cells maintain energy homeostasis during DNA repair [85,86].

The NRF2 transcription factor coordinates both antioxidant responses and metabolic reprogramming following DNA damage. NRF2 activation enhances NADPH production through multiple mechanisms, including upregulation of glucose-6-phosphate dehydrogenase and malic enzyme [87]. This serves the dual purpose of supporting both antioxidant defense and nucleotide synthesis required for DNA repair. The crosstalk between NRF2 and the DNA damage response is further evidenced by its interaction with regulatory proteins like BRCA1 [88].

## 6. DDR–Metabolism Crosstalk in Disease, Therapeutic Implications, and Challenges

Proper coordination between DNA damage responses and cellular metabolism is essential for maintaining health. Dysregulation of this crosstalk has been implicated in various pathological conditions, including cancer, neurodegenerative diseases, and aging [89,90]. Moreover, elucidating these interactions may unveil new therapeutic targets and strategies for treating these conditions [91].

### 6.1. Disease Implications

Cancer represents a prime example where DDR–metabolism crosstalk plays a crucial role. Cancer cells often exhibit both DNA repair deficiencies and metabolic alterations. For instance, *BRCA1*-deficient breast cancers show not only impaired homologous recombination but also significant metabolic rewiring, including increased glucose uptake and elevated oxidative stress [92]. The accumulation of DNA damage in cancer cells can trigger adaptive metabolic responses that contribute to therapy resistance [10]. Interestingly, these metabolic adaptations can become a double-edged sword: while they help cancer cells survive initial DNA damage, they may also create specific vulnerabilities that can be therapeutically exploited [93]. Studies have shown that these vulnerabilities often involve specific combinations of defects in DNA repair pathways and redox regulation systems [94].

In neurodegenerative diseases, the connection between DNA repair and metabolism becomes particularly relevant due to neurons’ high energy demands and limited regenerative capacity. Defects in DNA repair proteins like ATM lead to both accumulated DNA damage and mitochondrial dysfunction, contributing to neurodegeneration [95]. Studies in Alzheimer’s disease models have shown that DNA damage accumulation leads to metabolic alterations that exacerbate neuronal dysfunction [96]. This metabolic dysregulation can create a destructive cycle where impaired DNA repair and compromised metabolism mutually reinforce each other.

During aging, the relationship between DNA repair efficiency and metabolic health becomes increasingly important. Age-related decline in DNA repair capacity correlates with metabolic dysfunction and increased oxidative stress [97]. The NAD+ depletion that occurs with aging has been shown to impair both DNA repair capacity and metabolic homeostasis, creating a vicious cycle that accelerates aging-related pathologies [98].

### 6.2. Therapeutic Implications

Understanding the DDR–metabolism crosstalk has opened new therapeutic avenues. The exploration of synthetic lethal interactions between DNA repair and metabolic pathways has emerged as a promising approach for cancer treatment. For example, combining DNA repair inhibitors and metabolic inhibitors, such as PARP inhibitors with platinum-based agents, have shown promising results in treating cancers with DNA damage response (DDR) deficiencies [99,100]. The recognition that DNA repair processes can induce oxidative stress has led to the development of approaches that target both repair pathways and redox balance. Studies in yeast have identified strong synthetic lethal interactions between antioxidant genes and various DNA repair components. Specifically, *SOD1* and *TSA1* mutants show synthetic lethality with genes involved in homologous recombination (*RAD50*, *RAD52*) and post-replication repair (*RAD6*, *RAD18*) pathways [101,102]. Based on these findings, SOD1 inhibition was proposed as a novel therapeutic approach for colorectal cancers with *RAD54B* defects [103]. Similar synthetic lethal relationships were found in *BLM*- and *CHEK2*-deficient colorectal cancer cells, which were selectively killed by *SOD1* silencing [104]. The efficacy of targeting DNA repair-deficient cancer cells through ROS modulation can be further enhanced through combination approaches. For example, the selective killing of *RAD54B*-deficient colorectal cancer cells by PARP1 inhibitors is enhanced by silencing *SOD1* [105]. This suggests potential for developing more potent combinatorial chemotherapy regimens that exploit multiple synthetic lethal relationships simultaneously.

The mechanistic basis for these synthetic lethal interactions involves complex interplay between ROS and DNA repair pathways. Loss of antioxidant genes like *SOD1*, *TSA1*, or their upstream transcription factors YAP1 and SKN7 results in increased formation of Rad52 foci, indicating active DNA repair sites [106]. Importantly, reducing oxygen metabolism by growing cells in anaerobic conditions suppresses the lethality of double mutants like *tsa1 rad51*, *tsa1 rad6*, and *tsa1 mre11*, demonstrating that increased intracellular ROS directly contributes to synthetic lethality [107].

Despite these promising findings, several significant challenges remain in translating DNA repair-redox synthetic lethality to clinical applications. One major difficulty is that synthetic lethal interactions determined from high-throughput analysis are not always reliable and require further empirical testing. For instance, while initial screens suggested synthetic lethality between *rad51* and *sod1* in yeast, direct tetrad dissection revealed that the double mutant is viable, though showing increased sensitivity to ROS-inducing agents [108]. The conservation of synthetic lethal interactions between model organisms and humans is also variable and depends on specific genes, pathways, and cellular processes.

Additionally, synthetic lethal interactions can be highly context-dependent, affected by intracellular physiological status, microenvironment conditions, and accompanying drug treatments [109]. The metabolic plasticity of cancer cells can lead to resistance mechanisms [110,111]. This has been demonstrated in studies examining SOD1-based therapies, where despite initial effectiveness, cells can develop adaptive responses [103]. Furthermore, the systemic effects of targeting both DNA repair and metabolism need careful consideration, as demonstrated by the oxidative stress induced by many chemotherapeutic agents in normal tissues [10,112,113]. The therapeutic strategies must account for the complex relationship between DNA repair capacity and cellular redox state. For instance, some cancer cells may be particularly vulnerable to redox modulation due to deficiencies in specific DNA repair pathways [88].

Several key challenges remain in exploiting DDR–metabolism crosstalk therapeutically, including the need to understand the temporal dynamics of metabolic responses to DNA damage, as well as developing methods that specifically target disease-relevant metabolic pathways while sparing normal tissue. Additionally, identifying reliable biomarkers for patient stratification in metabolism-targeted therapies and devising strategies to prevent or minimize therapy-induced oxidative stress remain crucial for improving the therapeutic outcome. Addressing these challenges will be essential for optimizing DDR–metabolism-based therapies (a summary of the implications of DDR–metabolism crosstalk in disease and therapy is presented in Table 1).

## 7. Future Perspectives and Conclusions

The intricate relationship between DNA damage response and cellular metabolism represents a rapidly evolving field with significant implications for both basic research and therapeutic applications. While substantial progress has been made in understanding this crosstalk, several critical questions and challenges remain to be addressed.

First, the temporal dynamics of metabolic adaptations in response to DNA damage need further investigation. Specifically, how metabolic changes are coordinated with the different phases of the DNA damage response remains unclear, and understanding the precise timing of these events is essential for developing targeted interventions. Second, the spatial organization of metabolic resources during DNA repair is not well understood. The recruitment of metabolic enzymes to specific damage sites and how these processes are compartmentalized within cells require detailed study. Finally, cell-type specificity presents a major challenge, as different tissues may rely on distinct metabolic pathways to facilitate DNA repair. Understanding these tissue-specific requirements will be essential for designing therapeutic strategies that are tailored to the unique metabolic needs of different cell types.

Recent technological advances are opening new avenues for investigating DDR-metabolism interactions. The development of real-time metabolic sensors offers unprecedented opportunities to monitor metabolic changes during DNA repair with high temporal and spatial resolution [114,115,116,117,118]. CRISPR-based screening approaches have enabled systematic investigation of genetic interactions between DDR and metabolic pathways [119,120,121,122]. These tools will be crucial for unraveling the complex regulatory networks that coordinate DNA repair and metabolism.

### Concluding Remarks

The past decade has witnessed remarkable progress in understanding how cells coordinate DNA repair with metabolic adaptation. This crosstalk emerges as a fundamental aspect of cellular homeostasis, with implications ranging from normal physiology to disease treatment. The oxidative stress generated during DNA repair represents both a challenge and an opportunity for therapeutic intervention. Moving forward, integrating our understanding of DDR–metabolism crosstalk with emerging technologies and therapeutic strategies will be crucial for developing more effective and targeted treatments for cancer and other diseases characterized by genome instability.

## Figures and Tables

**Table 1:** DDR–metabolism crosstalk in disease and therapy.

Disease Context	Crosstalk Manifestations	Therapeutic Implications
Cancer	PARP hyperactivation → NAD+ depletion → metabolic crisis	Combined targeting of DNA repair and ROS regulation pathways
	DNA damage-induced ROS → metabolic rewiring	Exploitation of synthetic lethality between repair deficiency and redox regulation
	ATP/NAD+ availability affecting repair pathway choice	NAD+ metabolism modulation in repair-deficient cells
Neurodegeneration	ATM deficiency → mitochondrial dysfunction → impaired repair	NAD+ supplementation strategies
	DNA damage → NAD+ depletion → compromised SIRT1 activity	Combined targeting of repair and metabolic pathways
	Repair-induced oxidative stress → amplified damage	Antioxidant approaches in repair-deficient contexts
Therapeutic Resistance	Metabolic adaptation to DNA repair inhibition	Sequential or combination therapy approaches
	ROS-mediated feedback loops between repair and metabolism	Biomarker-guided treatment strategies
	Compensatory metabolic pathway activation	Monitoring of metabolic adaptation

## Data Availability

Not applicable.
